# 1522. Penicillin plus ceftaroline against *Enterococcus faecalis* blood isolates lacking ampicillin plus ceftriaxone activity

**DOI:** 10.1093/ofid/ofac492.084

**Published:** 2022-12-15

**Authors:** Angella Nwoye, Jessica Bocanegra, Ruhma Khan, Zeel Shah, Jaclyn A Cusumano

**Affiliations:** Long Island University, Brooklyn, New York; Long Island University, Brooklyn, New York; Long Island University, Brooklyn, New York; Long Island University, Brooklyn, New York; Long Island University, Brooklyn, New York

## Abstract

**Background:**

*Enterococcus faecalis* infective endocarditis standard of care treatment, ampicillin plus ceftriaxone, unfortunately has been unable to decrease mortality rates and additionally increases patient risk for vancomycin-resistant enterococci (VRE). Recent data also demonstrates decreased ampicillin plus ceftriaxone activity in *E. faecalis* isolates with an elevated penicillin MIC of 4 mcg/mL. Penicillin plus ceftaroline is a promising alternative but limited data is available to support.

**Methods:**

Seven *E. faecalis* clinical blood isolates and one wild-type *E. faecalis* isolate (i.e., JH2-2) were tested against penicillin plus ceftaroline utilizing 24-hour in vitro time-kill assays. All isolates were penicillin susceptible with minimum inhibitory concentrations (MIC) of either 2 (n=4) or 4 (n=4) mcg/mL, and ceftaroline MICs ranged from 1 to 8 mcg/mL. Penicillin and ceftaroline were tested at subinhibitory concentrations (0.25x and 0.5xMIC) as monotherapy and in combination. Antibacterial activity was described as bactericidal or bacteriostatic, defined as a ≥ 3-log_10_ colony-forming units (CFU)/mL or < 3-log_10_ CFU/mL decrease from initial inoculum, respectively. Synergy was defined as a ≥ 2-log_10_ decrease in CFU/mL at 24 hours from the most active single agent.

**Results:**

Penicillin and ceftaroline monotherapy did not demonstrate any antibacterial activity, except for ceftaroline 0.5xMIC where four isolates demonstrated bacteriostatic activity (Table 1). Penicillin plus ceftaroline was synergistic in all isolates except JH2-2 (penicillin MIC of 2 mcg/mL) due to the bacteriostatic activity of ceftaroline alone. Synergistic and bactericidal activity was also more frequently observed in isolates with a penicillin MIC of 4 mcg/mL with penicillin plus ceftaroline than ampicillin plus ceftriaxone.

**Conclusion:**

Penicillin plus ceftaroline demonstrates synergistic activity against clinical *E. faecalis* blood isolates including isolates with a penicillin MIC of 4 mcg/mL, where ampicillin plus ceftriaxone less frequently demonstrated synergy. Further research is warranted to support utilization in patients with *E. faecalis* infective endocarditis.

**Disclosures:**

**Jaclyn A. Cusumano, PharmD, BCIDP**, Shionogi: Advisor/Consultant.

**Table 1.**
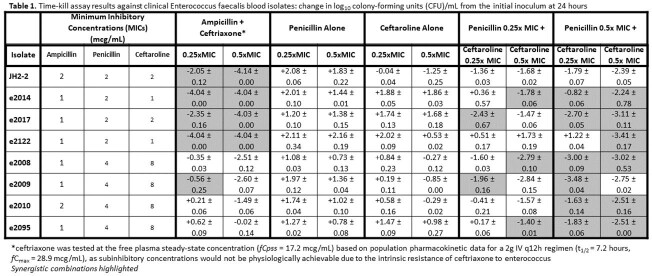
Time-kill assay results against clinical Enterococcus faecalis blood isolates: change in log10 colony-forming units (CFU)/mL from the initial inoculum at 24 hours

